# Temperament, Executive Functioning, and Anxiety in School-Age Children Who Stutter

**DOI:** 10.3389/fpsyg.2019.02244

**Published:** 2019-10-04

**Authors:** Mónica Soares Rocha, J. Scott Yaruss, Joana R. Rato

**Affiliations:** ^1^Institute of Health Sciences, Catholic University of Portugal, Lisbon, Portugal; ^2^Department of Communicative Sciences and Disorders, Michigan State University, East Lansing, MI, United States; ^3^Centre for Interdisciplinary Research in Health, Catholic University of Portugal, Lisbon, Portugal

**Keywords:** temperament, executive functions, anxiety, stuttering, school-age children

## Abstract

The purpose of this study was to examine temperament dimensions, executive functioning ability, and anxiety levels in school-age children who stutter and their non-stuttering peers. Participants were 100 Portuguese children aged 7 to 12 years (*M* = 9.13; *SD* = 1.70), including 50 children who stutter and 50 children who do not stutter. Analyses, which were performed separately for younger and older participants, sought to identify correlations between key variables. Temperament was evaluated through a parent questionnaire, executive functioning was evaluated through children’s responses on a performance test, and anxiety level was assessed through a self-perception scale. On the temperament measure, comparisons between children who stutter and their non-stuttering peers revealed that older children who stutter exhibited significantly higher scores on the Anger/Frustration, Impulsivity, and Sadness subscales, and lower averages on the Attention/Focusing, Perceptual sensitivity, and Soothability/Falling Reactivity subscales. On the executive functioning task, comparisons revealed that the group of younger children who stutter exhibited significantly higher average execution times than their non-stuttering peers. There were no statistically significant differences in anxiety between children who stutter and children who do not stutter, and there were no statistically significant correlations between temperament factors and measures of executive functioning. Children who stutter experienced lower ability to orient attention and greater emotional reactivity compared with their non-stuttering peers. Significant correlations were found between executive functioning and age and among the temperament factors themselves. These results, which support the need for a multidimensional view of stuttering, were interpreted in the context of the Dual Diathesis – Stressor model. Findings indicate that temperament and executive functioning abilities may contribute to the development of stuttering.

## Introduction

### Temperament

Temperament is an overarching term for a collection of traits that are assumed to be biologically determined and related to individual differences in reactivity and self-regulation (Rothbart et al., 2000; Jones et al., 2014).

Temperament can develop over time (Goldsmith et al., 1987) and be influenced by environmental interactions (Eggers et al., 2010). According to Rothbart and colleagues, “constitutional” factors are associated with genes and environment, “reactivity” is related to sensory response systems, and “self-regulation” relates to the process that can facilitate or inhibit reactivity (Rothbart et al., 2000). Thomas and Chess (1996) described nine temperament dimensions: “Activity Level,” “Rhythmicity,” “Approach/Withdrawal,” “Adaptability,” “Threshold of Responsiveness,” “Intensity of Reaction,” “Quality of Mood,” “Distractibility,” “Attention Span,” and “Persistence.” The authors related temperament to the expression of a particular behavior. Children’s and adults’ intrinsic motivations and abilities for a specific behavior can be mediated by aspects of their temperament, such as their activity level, their adaptability, and their persistence (Goldsmith et al., 1987). Some authors have connected temperament differences in children who stutter with their susceptibility to begin, continue, or recover from stuttering (Conture, 2001; Guitar, 2014; Ambrose et al., 2015). Specifically, studies have suggested that children with a sensitive temperament may have neural vulnerabilities that cause them to be more likely to develop stuttering (Guitar, 2014).

Findings regarding temperament in children who stutter have been inconsistent. Therefore, it is not yet possible to draw firm conclusions about differences in temperament between children who stutter and their non-stuttering peers. Still, there is an increasing literature reporting a propensity for a more reactive and sensitive temperament in children who stutter (Embrechts et al., 2000; Felsenfeld et al., 2000; Karrass et al., 2006; Eggers et al., 2010; Alm, 2014; Ambrose et al., 2015), and there is indication that more reactive and sensitive children tend to respond more strongly to disruptions in speech fluency (Walden et al., 2012).

Temperamental characteristics in preschool children that have been shown to contribute to stuttering include difficulty concentrating on tasks (Embrechts et al., 2000; Anderson et al., 2003), and low frustration tolerance (Reilly et al., 2009; Eggers et al., 2010; Druker et al., 2019). According to Spaulding et al. (2008), tasks dependent on sustained selective attention may be influenced by limited processing resources and situational demands. It is also known that attentional control plays an important role in children’s ability to manage and regulate their emotions (Blair and Ursache, 2011). Several studies have reported that preschool children who stutter are prone to have difficulty adapting to new objects and situations (Embrechts et al., 2000; Anderson et al., 2003; Howell et al., 2004; Schwenk et al., 2007; Reilly et al., 2009; Eggers et al., 2010; Hollister, 2015) and have a tendency toward greater negative affect (Embrechts et al., 2000; Ntourou et al., 2013) and negative mood (Howell et al., 2004). Experimental studies of the temperament of preschool children who stutter have revealed a tendency for impulsivity (Schwenk et al., 2007; Eggers et al., 2010) and for lower self-regulation, or the ability to regulate emotional behaviors (Johnson et al., 2010; Ntourou et al., 2013).

While studies of temperament in preschool children and adults who stutter have revealed notable differences compared to peer groups who do not stutter (e.g., Reilly et al., 2009; Ntourou et al., 2013; Ambrose et al., 2015; Smith and Weber, 2017), temperament studies involving school-age children are more rare (Oyler, 1996; Nicholas et al., 2015). Those that have been conducted have shown that children of this age who stutter tend to be more sensitive and withdrawn than their non-stuttering peers (Fowlie and Cooper, 1978). There is a need to further research temperament in school-age children in order to understand the changes that arise throughout a child’s development. In the same way that some studies conclude that young children and adults who stutter exhibit certain temperament characteristics, it is important to determine whether these characteristics maintain or otherwise change during the school-age years and how they contribute to cognitive development (Singer and Fagen, 1992).

### Executive Functioning

The role of EF in childhood stuttering has been a subject of increased attention in recent years (Ntourou et al., 2013; Jones et al., 2014). EF is a term used to describe a diverse set of cognitive skills needed to perform activities that require planning and monitoring of intentional behaviors that allow individuals to interact with the world in an adaptive and appropriate way (Diamond, 2013). Researchers have highlighted three basic components of EF: inhibition, the ability to suppress a prepotent response; working memory, which implies an information-updating process; and shifting, the ability to shift between tasks or mental sets and is an important aspect of executive control (Miyake et al., 2000). Despite some inconsistencies in findings across studies, several studies have shown that children who stutter, especially in earlier ages, have a tendency to be less successful in maintaining attention than their typically fluent peers (Heitmann et al., 2004; Kaganovich et al., 2010; Costelloe et al., 2015; Eichorn et al., 2017). Children who stutter are also prone to be less able to select information from sensory input (Eggers et al., 2012), more likely to exhibit impulsivity (Eggers et al., 2013), and more likely to have greater concern about their performance (Eichorn et al., 2017).

Symptoms similar to those seen in children with attention deficit disorders have been identified in some children who stutter (Anderson et al., 2003; Druker et al., 2019); however, studies related to the incidence of attention deficit disorders are not conclusive and have been performed with a limited sample size (Riley and Riley, 2000; Donaher and Richels, 2012). Children who stutter tend to perform less well than their peers in working memory (Anderson and Wagovich, 2010; Oyoun et al., 2010), inhibitory control (stroop-like tasks), and attentional focusing, as indicated through parent ratings (Wolfe and Bell, 2004; Bajaj, 2007). Difficulties related to inhibitory control and attentional focusing are especially evident in studies that use parent-report questionnaires (Ofoe et al., 2018).

Cognitive processes described above are closely linked to emotional regulation (Sudikoff et al., 2015) and can influence the experience of anxiety (Craske et al., 2009).

### Anxiety

Anxiety is a general term for an individual’s emotional struggle that combines nervousness, fear, apprehension, and worrying (Craske et al., 2009). According to some authors (e.g., Craig and Hancock, 1996; Craig et al., 2003; Ezrati-Vinacour and Levin, 2004; Craig and Tran, 2014), anxiety can be divided into *trait* anxiety (related to stable anxious baseline characteristics) and *state* anxiety (related to transitory conditions due to unpleasant emotional arousal with a tendency to appear when people have to cope with demanding situations). People who stutter often struggle with *state anxiety*, since anxiety will likely become a secondary effect of living with stuttering condition rather than being a static condition (Alm and Risberg, 2007; Messenger et al., 2015). Also, according to Samochiş et al. (2011), increased anxiety is a normal reaction to the physical aspects of stuttering. Nevertheless, some studies have not supported a relationship between anxiety and stuttering or have found little significant differences (e.g., Andrews and Harris, 1964; Hedge, 1972; Andrews et al., 1983; Cox et al., 1984; Peters and Hulstijn, 1984; Craig and Hancock, 1996). Currently, the occurrence of anxiety in children who stutter is still a subject of debate (Alm and Risberg, 2007; Manning and Beck, 2013; Alm, 2014; Craig, 2014; Smith et al., 2014). Even in the literature that does support the existence of anxiety in children, the age at which anxiety symptoms begin to appear has not yet been identified. Specifically, the studies linking anxiety to preschool-age children have shown no differences between children who stutter and non-stuttering peers on anxiety measures and salivary cortisol levels (van der Merwe et al., 2011). Some studies have found significantly higher anxiety symptoms in school age children who stutter, ages 7 to 12 (e.g., Iverach et al., 2011), and other studies have reported the same for children from 10 and up (Davis et al., 2007; Mulcahy et al., 2008; McAllister et al., 2015; Iverach et al., 2017). Nevertheless, other studies have not found any trend toward elevated anxiety in school age children (Andrews and Harris, 1964; Craig and Hancock, 1996; Ortega and Ambrose, 2011). Some evidence suggests that the levels of anxiety tend to increase over time and can exceed normal values in adolescence and adulthood (Mulcahy et al., 2008). Still, the meaning of these findings is unclear, and according to Messenger et al. (2015), adolescents who stutter may try to present themselves positively to hide their true concerns about stuttering. This lack of consistency suggests the existence of other variables that might affect the development of anxiety.

### Temperament, EF, Anxiety, and the Dual Diathesis-Stressor Model

To date, no studies have simultaneously considered the relationship between temperament, EF, and anxiety in children who stutter, even though all of these factors are believed to play a role in stuttering. Because of the relationship between anxiety, temperament, and EF (Nigg, 2000), considering these factors in concert will help to elucidate how these issues relate to the development and experience of stuttering.

There is already a large body of empirical evidence suggesting a strong concurrent relationship between temperament characteristics and executive functioning (EF) (Simonds, 2006; Sudikoff et al., 2015). According to Affrunti and Woodruff-Borden (2015) the expression of temperament may be influenced by executive functioning. Temperament also includes behavioral aspects, such as approach and withdrawal, as well as attentional processes, including orientation maintenance and executive control. Together, these abilities are the building blocks of the development of self-regulation (Rothbart and Hwang, 2002). Studies of cognitive development have shown that attention control, inhibition of inappropriate behavior, decision making, and other cognitive processes that occur in emotionally demanding contexts, are strongly supported by EF (Gupta et al., 2011).

Research has further identified temperamental characteristics and cognitive abilities as predictors of anxiety (Kefalianos et al., 2012). Environmental factors can be part of these dynamic interactions and, together with temperamental characteristic and cognitive abilities, influence how children deal with stuttering. Because temperament characteristics and EF abilities may contribute to a child’s likelihood of responding to experiences in a particular way, the involvement of temperament and EF in the development of stuttering can be described in terms of the dual diathesis-stressor (DD-S) model (Walden et al., 2012). The DD-S model proposes that endogenous abilities of children who stutter interact in a dynamic way with exogenous contexts (stressors). In line with this model, temperament and EF characteristics can be seen as a diathesis that can be triggered by a stressor, transforming a predisposition to an actual emotional response in a particular situation. As applied to stuttering, the theory suggests that a child’s endogenous characteristics related to temperament, anxiety, and EF, may be affected by exogenous stressors that may increase (or decrease) the frequency of stuttering. Importantly, exogenous contexts (stressors) can activate cognitive and affective processes and pushing the autonomic nervous system out of homeostasis, thereby increasing the emotional response (Walden et al., 2012). This imbalance can translate into anxiety and other signs of dysregulation (Craske et al., 2009).

The present study was designed to address the literature gap on the research of temperament, EF, and anxiety jointly, comparing school-age children who stutter and non-stuttering peers. The combination of these three aspects can give us further information about the interaction between emotional and cognitive factors. Moreover, the DD-S model, which focuses the interaction between intrinsic and external factors and how they may change over time, highlights the need to concurrently consider factors such as temperament, EF, and anxiety. Taken together, these factors can provide more clues about the onset, development, and possible persistence of stuttering during childhood. A better understanding of such relationships may help clinicians understand how stuttering affects children, and this understanding may contribute to the development of more effective and personalized treatment programs.

## Materials and Methods

### Participants

Participants were 100 Portuguese children, 50 children who stutter (“S” Group) and 50 age-matched children who do not stutter (“N” Group), ages 7 to 12 years old. The Stuttering Severity Instrument – 4^th^ Edition (SSI-4) (Riley, 2009) was used to confirm and diagnose stuttering.

Table 1 shows the demographic characteristics of the participants. The sex ratio of participants who stutter was 2.6 males to each female; for participants who do not stutter, it was 0.8 males to each female. This sex ratio for children who stutter is consistent with previous literature (Craig et al., 2002; Yairi and Ambrose, 2005).

**TABLE 1:** Demographic characteristics of the participants (children who stutter = 50; children who do not stutter = 50).

**Group**	**Children who**	**Children who**	**Total**
	**stutter**	**do not stutter**	
Age mean (SD)	9.10 (1.73)	9.16 (1.68)	9.13 (1.70)
Sex (M/F)	36/14	22/28	58/42
	(72%/28%)	(44%/56%)	(58%/42%)
**Education level (n)**			
1^st^ grade	8(16%)	3(6%)	11(11%)
2^nd^ grade	11(22%)	10(20%)	21(21%)
3^rd^ grade	9(18%)	8(16%)	17(17%)
4^th^ grade	7(14%)	14(28%)	21(21%)
5^th^ to 7^th^ grade	15(30%)	15(30%)	30(30%)
**Treatment (n)**			
Without treatment	14(28%)	–	–
Speech therapy	11(22%)	–	–
Waiting or initiating	14(28%)	–	–
Previous therapy	11(22%)	–	–

In order to explore developmental differences, the participants who stutter (*n* = 50) and their non-stuttering peers (*n* = 50) were grouped according to age: younger children (7–9 years old; *M* = 7.92; *SD* = 0.81) and older children (10–12 years old; *M* = 10.95; *SD* = 0.82).

The cutoff age point for the two groups in this study was based on the development and important changes that take place during this period, in which previously acquired learning is consolidated and new intellectual, psychological and social acquisitions arise (Blake and Pope, 2008). In addition, this age group distinction corresponds to the first two education cycles in Portugal: the first cycle includes the first 4 years of school (about 7–9 years old) and the second cycle includes the 5^th^ and 6^th^ grades (about 10–12 years old). Depending upon a child’s birth date, however, it is possible to find children in the 7^th^ grade who are 12 years old. Pre-school education in Portugal is intended for children between 3 and 6 years old; from the age of 13, Portuguese children are usually in high school (Alarcão et al., 2009).

Inclusion and exclusion criteria ensured that children did not exhibit any neurological or psychiatric impairment, learning disorder, or history of head injury or seizures. The sample was chosen by convenience: participants who stutter were recruited from speech-language therapists and through referral of school teachers; participants who do not stutter were recruited in some schools attended by their stuttering peers. All children were monolingual speakers of Portuguese.

When the study was performed, 22% of the children who stutter were in speech therapy, 22% had previous speech therapy, and 28% were waiting for therapy or just initiating speech therapy. The children who were in therapy at the time of data collection had been in treatment between 1 to 96 months (*M* = 9.30 mos.; *SD* = 19.38 mos.). Children who had previous therapy had received between 3 and 48 months of treatment (*M* = 13.28 mos.; *SD* = 12.99 mos.).

### Materials

The SSI-4 (Riley, 2009) was used along with the Portuguese story, “*A história do rato Artur*” (Guimarães, 2007). “Rato Artur” story has been used in several Portuguese studies (e.g., Guimarães and Abberton, 2005; Silvestre, 2009; Silvestre et al., 2011), because it has a high test–retest consistency and is phonetically balanced. This has been interpreted to indicate that is close to spontaneous discourse (Moon et al., 2012). Eight of the 7-year-old participants had difficulties reading the story, so only the SSI-4 plates were used for those participants.

The parents provided information about socio-demographic background, and the child’s stuttering via a checklist created for this study. Table 1 shows information about children; Table 2 shows information about parents’ sex, age, education level, and family history of stuttering.

**TABLE 2:** Demographic characteristics of parents (parents of children who stutter = 50; parents of children who do not stutter = 50).

**Group**	**Parents of children**		**Parents of children**		**Total**	
	**who stutter**		**who do not stutter**			
Age mean (SD)	42.26 (4.82)		39.60 (4.34)		40.93 (4.76)	
Sex (M/F)	6/44 (6%/88%)		3/47 (6%/94%)		9/91/ (9%/91%)	
**Family history of stuttering (n)**						
Yes	30 (60%)		–		–	
No	20 (40%)		–		–	

**Education level (n)**	**Mother**	**Father**	**Mother**	**Father**	**Mother**	**Father**

1–4 years	0(0%)	0(0%)	0(0%)	2(4%)	0(0%)	2(2%)
5–6 years	0(0%)	0(0%)	2(4%)	0(0%)	2(2%)	0(0%)
7–9 years	5(10%)	6(12%)	2(4%)	7(14%)	7(7%)	13(13%)
10–12 years	8(16%)	10(20%)	15(30%)	19(38%)	23(23%)	29(29%)
Graduation	32(64%)	30(60%)	27(54%)	21(42%)	59(59%)	51(51%)
Master	3(6%)	4(8%)	3(6%)	0(0%)	6(6%)	4(4%)
Ph.D.	2(4%)	0(0%)	1(2%)	1(2%)	3(3%)	1(1%)

#### Temperament

The Temperament in Middle Childhood Questionnaire (TMCQ) (Simonds and Rothbart, 2004) is a parent-reported, paper-and-pencil measure that evaluates temperament in middle childhood (7–10 years old). It consists of 157 questions that examine 17 dimensions of temperament: (1) Activity Level, (2) Affiliation, (3) Anger/Frustration, (4) Assertiveness/Dominance, (5) Attention Focusing, (6) Discomfort; (7) Fantasy/Openness, (8) Fear, (9) High Intensity Pleasure, (10) Impulsivity, (11) Inhibitory Control, (12) Low Intensity Pleasure, (13) Perceptual Sensitivity, (14) Sadness, (15) Shyness, (16) Soothability/Falling Reactivity, (17) Activation Control (see Table 3). Answers are obtained by parents rating their children on five-point Likert scales ranging from “Almost always untrue” to “Almost always true,” with the option of “Does not apply.”

**TABLE 3:** TMCQ scale (Simonds and Rothbart, 2004) descriptions and sample items.

**TMCQ scale**	**Definition**
Activity level	Level of gross motor activity including rate and extent of locomotion.
Affiliation	The desire for warmth and closeness with others, independent of shyness or extraversion.
Anger/frustration	Amount of negative affect related to interruption of ongoing tasks or goal blocking.
Assertiveness/dominance	Tendency to speak without hesitation and to gain and maintain control of social situations.
Attentional focusing	Tendency to maintain attentional focus upon task-related channels.
Discomfort	Amount of negative affect related to sensory qualities of stimulation, including intensity, rate or complexity of light, movement, sound, and texture.
Fantasy/openness	Active imagination, aesthetic sensitivity, intellectual curiosity.
Fear	Amount of negative affect, including unease, worry or nervousness related to anticipated pain or distress and/or potentially threatening situations.
High intensity pleasure	Amount of pleasure or enjoyment related to situations involving high stimulus intensity, rate, complexity, novelty, and incongruity.
Impulsivity	Speed of response initiation.
Inhibitory control	The capacity to plan and to suppress inappropriate approach responses under instructions or in novel or uncertain situations.
Low intensity pleasure	Amount of pleasure or enjoyment related to situations involving low stimulus intensity, rate, complexity, novelty, and incongruity.
Perceptual sensitivity	Amount of detection of slight, low intensity stimuli from the external environment.
Sadness	Amount of negative affect and lowered mood and energy related to exposure to suffering, disappointment, and object loss.
Shyness	Slow or inhibited approach in situations involving novelty or uncertainty.
Soothability/falling reactivity	Rate of recovery from peak distress, excitement, or general arousal.
Activation control	The capacity to perform an action when there is a strong tendency to avoid it.

Through the TMCQ, it is possible to identify reactivity/sensitivity and self-regulation characteristics. For example, the TMCQ scales such as Anger/frustration are connected to reactivity, whereas scales such as Inhibitory control are more related to self-regulation (Eggers et al., 2013). For example, young children may become angry and impulsive when their goals are hindered. This might occur when they have to wait for something they want (Rothbart et al., 2001).

Of the 17 dimensions of temperament that are part of the instrument, 13 dimensions derive from the well-validated Children’s Behavior Questionnaire (CBQ: Rothbart et al., 2001), which has been used in several studies to investigate the relationship between temperament and stuttering (e.g., Eggers et al., 2010; Ambrose et al., 2015). In Simonds (2006), the TMCQ was shown to have good internal consistency reliability (Cronbach’s alpha ranged from 0.62 to 0.83) and acceptable agreement between self-report and parent report (Pearson’s r ranged from −0.02 to 0.50).

The questionnaire was translated to European Portuguese for this study (Rocha and Rato, 2017).

#### Executive Functioning

Children were assessed using the Portuguese version of the Children’s Color Trails Test (CCTT), a neuropsychological paper-and-pencil test of EF (Pinto, 2008). The CCTT measures sustained visual attention, sequencing, psychomotor speed, and cognitive flexibility. It is intended for ages 8 to 16, though the authors have reported success with children as young as 7 years old (Llorente et al., 2003). The test includes two parts (CCTT-1 and CCTT-2), each involving one trial and one experimental task. In CCTT1, the child must connect the numbers from 1 to 25 following a correct sequence as quickly as possible. In CCTT2, the child must repeat the task from CCTT1 but with a color alternation. In this task, the child still connects the numbers from 1 to 25. This time, however, each number is repeated in different colors (i.e., there are yellow numbers and pink numbers), and the child must be sure to follow the numerical order even when it changes between yellow and pink (Llorente et al., 2003).

The results of both parts of this test consist of: (a) time (in seconds) that the child takes to complete the tasks, (b) the number of times almost failed (the failures), (c) the number of errors, and (d) the number of warnings (when a child makes a mistake, the examiner advises him or her to start the test again from the last correct circle).

CCTT has been increasingly used around the world (e.g., Koo and Min, 2008; Pinto, 2008; Llorente et al., 2009; Konstantopoulos et al., 2015) for the assessment of children with neurological and psychiatric disorders such as language disabilities (e.g., Williams et al., 1995), attention deficit/hyperactivity disorder (e.g., Kennel et al., 2010; Cho et al., 2011), and other conditions (Llorente et al., 2003). CCTT is based on the Trail Making Test, which assess speeded visuomotor tracking. Research has shown discriminant validity and sensitivity across cultures (Williams et al., 1995). The CCTT is expected to have the same validity as the Trail Making in the assessment of children with several disorders (Williams et al., 1995). In a study with 70 children diagnosed with attention deficit and hyperactivity disorder, CCTT exhibited appropriate test–retest reliability (Llorente et al., 2009).

#### Anxiety

The children also completed the Portuguese version of Multidimensional Anxiety Scale for Children (MASC), which examines the symptoms of anxiety in children and adolescents ages 7 to 19 years. It contains 39 questions, with four-point Likert scale responses (March et al., 1997; Matos et al., 2012; Salvador et al., 2017). Items on this questionnaire are grouped into four factors: (a) Physical symptoms, (b) Social anxiety, (c) Separation anxiety, and (d) Harm avoidance (Wei et al., 2014). Participants are asked to score statements such as: “I get nervous if I have to do something in public,” choosing between: (a) “it is never or almost never true,” (b) “it is rarely true,” (c) “sometimes it is true,” and (d) “It is often true.”

The normative data for the MASC show that it is oriented mainly toward inherent characteristics (*trait* anxiety), though it is also influenced by transitory conditions and situations (*state* anxiety) (March et al., 1997). Decades of research confirm the robust features of the MASC. Several studies with general populations and with clinical populations have supported scale’s internal consistency, temporal stability, and convergent validity (Salvador et al., 2017). The original English version demonstrated good internal consistency (between 0.60 and 0.90), strong convergent/divergent validity, and strong test–retest reliability (March et al., 1997). The Portuguese version of the MASC has also been shown to be an adequate and reliable measure for self-assessment of anxious symptomatology, presenting reasonable psychometric characteristics in internal consistency, temporal stability, and validity (Salvador et al., 2017).

### Procedures

This study received full approval by the Ethics Committee of the Institute of Health Sciences of Universidade Católica Portuguesa (register number 34/2017). Prior to their participation in this study, parents signed a written informed consent for themselves and their children. Consent also included permission for the researcher to record the child and the right for participants to withdraw from the study at any time was clarified.

Children were assessed while parents completed the questionnaires. This was carried out in two sessions of approximately 30 min each.

All testing was conducted between December 2017 and May 2018. The SSI, MASC, and CCTT instruments were applied on different days and in a different order, to reduce potential order effects that might bias results.

#### Temperament

Temperament was assessed using the Portuguese version of the TMCQ, with the 157 original questions, distributed in 17 temperament dimensions (Simonds and Rothbart, 2004). After a brief explanation from the researcher, parents completed the TMCQ. This required approximately 20 min. In addition to researcher’s explanation, on the first page of the questionnaire parents could read instructions about the content of the questions and how to complete the form. After parents completed the questionnaire, the researcher scored the instrument according to the instructions.

#### Executive Functioning

For the EF assessment, the researcher presented and explained to the children how to perform the CCTT1, using the trial test. In both trial test and experimental test, children drew a line between the circles following a numerical order, as fast as they could; however, the CCTT1 trial test was performed with just 8 numbers. For the CCTT2 the procedures were similar, with the difference that children should switched between colors (after a yellow circle the child should drew a line toward a pink circle, following a numerical order). The researcher recorded 9 scores for each child. These scores corresponded to: the time that the child took to complete the tests for both CCTT1 and CCTT2, as well as the number of warnings, failures, and wrong answers (Number Sequencing and Color Sequencing) (Llorente et al., 2003).

#### Anxiety

For the anxiety assessment, the MASC questionnaire was presented to each child. Children were asked to read all the questions and to choose the best option for each. Children were informed about the importance of responding to all questions. For 7-years-old children, the MASC questions were read in full by the examiner.

After the children completed the questionnaire, the researcher summed the items for each factor, obtaining four final scores, corresponding to: (a) Physical symptoms, (b) Social anxiety, (c) Separation anxiety, and (d) Harm avoidance.

#### Data Analysis

Preliminary analyses were made in order to check the assumptions of homogeneity. Results for some variables were not normally distributed; however, with the *n* = 50 for each participant group, the central limit theorem suggests that parametric tests (*t*-test) would still be sufficiently robust to avoid deviations from normality. Two-sample *t*-tests were used to compare mean scores for the stuttering and non-stuttering groups for the temperament (TMCQ), EF (CCTT), and Anxiety (MASC) measures. These analyses were performed separately for younger and older participants. A multivariate analysis using principal component analysis (PCA) was performed in order to determine which variables were correlated and to summarize children characteristics in an ordination diagram. For the PCA analyses, younger and older children were separetely. This was done because of apparent differences between age groups. The use of PCA provided a dynamic view of the interaction among all of the variables, including age. To account for the large number of variables in the study (temperament, EF, anxiety, and age) only the variables that showed statistical significance in the t-tests were used in the PCA. Data analysis was completed using SPSS (Statistical Package for the Social Sciences – Version 24 for windows, IBM, Corp., Armonk, NY, United States).

## Results

### Younger Children Group

#### Temperament

No statistically significant differences were found between groups of children who stutter and their non-stuttering peers (*p* > 0.05) for any of the variables of temperament including: (1) Activity Level, (2) Affiliation, (3) Anger/Frustration, (4) Assertiveness/Dominance, (5) Attention Focusing, (6) Discomfort; (7) Fantasy/Openness, (8) Fear, (9) High Intensity Pleasure, (10) Impulsivity, (11) Inhibitory Control, (12) Low Intensity Pleasure, (13) Perceptual Sensitivity, (14) Sadness, (15) Shyness, (16) Soothability/Falling Reactivity, (17) Activation Control.

#### Executive Functioning

Group comparisons of the CCTT1 and the CCTT2 revealed that children who stutter exhibited significantly higher scores for execution time (CCTT1: *t*_(__48__.__75__)_ = 3.144, *p* = 0.003; CCTT2: *t*_(__52__.__27__)_ = 3.753, *p* < 0.001), as well as number of failures (CCTT1: *t*_(__38__.__23__)_ = 2.627, *p* = 0.012), number of warnings (CCTT1: *t*_(__52__.__47__)_ = 2.968, *p* = 0.005; CCTT2: *t*_(__53__.__71__)_ = 3.757, *p* < 0.001), number of sequencing errors (CCTT2: *t*_(__34__.__99__)_ = 3.337, *p* = 0.002), and color sequencing errors (CCTT2: *t*_(__49__.__31__)_ = 2.416, *p* = 0.020) (Table 4).

**TABLE 4:** Mean (M), standard deviations (SD) and *p*-values for the temperament, EF and anxiety performance tasks for group of younger children who stutter (*n* = 31; sex: M = 25; F = 6) and who do not stutter (*n* = 31; sex: M = 15; F = 16).

	**Children who stutter**		**Children who do not stutter**			
Scores	*M*	*SD*	*M*	*SD*	*t*	*p*
Activation control	3.185	0.442	3.326	0.493	–1.183	0.242
Activity level	3.632	0.755	3.794	0.709	–0.087	0.386
Affiliation	4.042	0.361	4.033	0.488	0.086	0.932
Anger/frustration	3.251	0.742	3.137	0.552	0.687	0.994
Assertiveness/dominance	3.122	0.646	3.300	0.597	–1.124	0.265
Attention/focusing	2.840	0.993	3.513	1.990	–1.686	0.097
Discomfort	2.819	0.669	2.481	0.669	1.993	0.051
Fantasy/openness	3.766	0.660	3.857	0.552	0.586	0.560
Fear	2.804	0.689	2.612	0.606	1.167	0.298
High intensity pleasure	3.058	0.651	2.998	0.624	0.373	0.711
Impulsivity	2.983	0.544	2.959	0.523	0.184	0.854
Inhibitory control	2.962	0.575	3.110	0.587	–1.000	0.322
Low intensity pleasure	3.256	0.655	3.477	0.629	–1.359	0.179
Perceptual sensitivity	3.091	0.835	3.206	0.692	–0.591	0.557
Sadness	2.700	0.452	2.713	0.586	–0.095	0.925
Shyness	2.792	0.811	2.651	0.852	0.664	0.509
Soothability/falling reaction	3.223	0.721	3.367	0.571	–0.831	0.410
CCTT1 time (sec)	86.308	33.943	64.032	20.107	3.144	0.003^∗∗^
CCTT1 number sequencing Errors	0.193	0.543	0.069	0.359	1.068	0.290
CCTT1 failures	0.548	0.961	0.065	0.359	2.627	0.012^∗^
CCTT1 warnings	1.677	1.558	0.677	1.045	2.968	0.005^∗∗^
CCTT2 time (sec)	161.420	46.582	123.677	31.061	3.753	< 0.001^***^
CCTT2 color sequencing errors	1.355	1.279	0.709	0.772	2.406	0.020^∗^
CCTT2 number sequencing Errors	0.419	0.620	0.032	0.120	3.337	0.002^∗∗^
CCTT2 failures	1.452	1.480	0.810	1.167	1.906	0.061
CCTT2 warnings	2.710	2.036	1.032	1.426	3.757	< 0.001^***^
Physical symptoms	6.258	4.885	7.267	5.836	0.733	0.467
Social anxiety	10.710	8.038	9.833	5.522	0.498	0.621
Separation anxiety	9.000	4.219	9.500	4.276	–0.460	0.647
Harm avoidance	19.774	4.566	19.700	4.276	0.065	0.948
Total score anxiety	45.420	15.000	46.267	14.694	–2.223	0.824

*^∗^*p* < 0.05, ^∗∗^*p* < 0.01, ^∗∗∗^*p* < 0.001.*

#### Anxiety

No statistically significant differences were found between groups of children who stutter and their non-stuttering peers (*p* > 0.05) for any of the variables of anxiety including: (1) Physical symptoms, (2) Social anxiety, (3) Separation anxiety, and (4) Harm avoidance, for each child.

### Older Children Group

#### Temperament

Statistically significant differences were found for several temperament factors (Table 5). Children who stutter scored lower than non-stuttering peers in Attention/Focusing (*t*_(__36__)_ = −3.526, *p* = 0.001), Perceptual Sensitivity (*t*_(__36__)_ = −2.411, *p* = 0.021), and Soothability/Falling Reactivity (*t*_(__36__)_ = −2.932, *p* = 0.006). Children who stutter scored higher than non-stuttering peers in temperament factors of Anger/Frustration (*t*_(__36__)_ = 2.801, *p* = 0.008), Impulsivity (*t*_(__36__)_ = 2.899, *p* = 0.006), and Sadness (*t*_(__36__)_ = 3.683, *p* = 0.001).

**TABLE 5:** Mean (M), standard deviations (SD) and *p*-values for the temperament, EF and anxiety performance tasks for group of older children who stutter (*n* = 19; sex: M = 11; F = 8) and children who do not stutter (*n* = 19; sex: M = 7; F = 12).

	**Children who stutter**		**Children who do not stutter**			
**Scores**	***M***	***SD***	***M***	***SD***	***t***	***p***
Activation control	3.049	0.467	3.221	0.406	–1.205	0.236
Activity level	3.872	0.694	3.806	0.800	0.271	0.788
Affiliation	4.126	0.449	4.171	0.412	0.320	0.751
Anger/frustration	3.335	0.614	2.807	0.546	2.801	0.008^∗∗^
Assertiveness/dominance	3.243	0.736	3.324	0.566	–3.81	0.071
Attention/focusing	2.644	0.644	3.552	0.920	–3.526	0.001^∗∗∗^
Discomfort	2.779	0.627	2.584	0.553	1.015	0.317
Fantasy/openness	2.916	0.814	3.840	0.536	–1.269	0.212
Fear	2.916	0.814	2.700	6.690	0.881	0.384
High intensity pleasure	3.084	0.576	2.783	0.655	1.501	0.142
Impulsivity	3.084	0.446	2.560	0.650	2.899	0.006^∗∗^
Inhibitory control	3.317	0.448	3.536	0.574	–1.312	0.198
Low intensity pleasure	3.264	0.476	3.435	0.572	–0.997	0.325
Perceptual sensitivity	3.307	0.567	3.722	0.491	–2.411	0.021^∗^
Sadness	3.036	0.553	2.415	0.485	3.683	0.001^∗∗∗^
Shyness	3.042	0.986	2.838	0.858	0.679	0.501
Soothability/falling reactivity	3.157	0.402	3.663	0.636	–2.932	0.006^∗∗^
CCTT1 time	51.745	15.000	52.790	19.472	–0.185	0.854
CCTT1 number sequencing errors	0.263	0.561	0.211	0.535	0.296	0.769
CCTT1 failures	0.158	0.375	0.000	0.000	1.837	0.074
CCTT1 warnings	0.632	1.065	0.211	0.419	1.604	0.118
CCTT2 times	97.9474	32.732	98.947	33.311	–0.093	0.926
CCTT2 color sequencing errors	0.579	1.610	0.421	0.838	0.379	0.707
CCTT2 number sequencing errors	0.000	0.000	0.000	0.000	0.073	0.943
CCTT2 failures	1.105	1.370	0.579	0.837	1.429	0.162
CCTT2 warnings	0.421	0.961	0.368	0.831	0.181	0.858
Physical symptoms	8.842	7.654	6.800	3.721	1.051	0.303
Social anxiety	10.263	7.001	11.526	4.937	0.642	0.525
Separation anxiety	9.474	6.040	8.526	4.033	0.569	0.573
Harm avoidance	17.947	4.972	18.158	4.375	–10.139	0.891
Total score anxiety	46.579	19.585	44.158	10.569	0.474	0.638

*^∗^*p* < 0.05, ^∗∗^*p* < 0.01, ^∗∗∗^*p* < 0.001.*

#### Executive Functioning

No statistically significant differences were found between groups of children who stutter and their non-stuttering peers (*p* > 0.05) for any of the variables of EF, including: (1) CCTT1 execution time, (2) CCTT1 number of sequencing errors, (3) CCTT1 number of failures, (4) CCTT1 number of warnings, (5) CCTT2 execution time, (6) CCTT2 number of color sequencing errors, (7) CCTT2 number of sequencing errors, (8) CCTT2 number of failures, (9) CCTT2 number of warnings.

#### Anxiety

As in the younger group, no statistically significant differences were found between groups of children who stutter and their non-stuttering peers (*p* > 0.05) for any of the variables of anxiety, including: (1) Physical symptoms, (2) Social anxiety, (3) Separation anxiety, and (4) Harm avoidance.

### Multivariate Analysis

**FIGURE 1:**
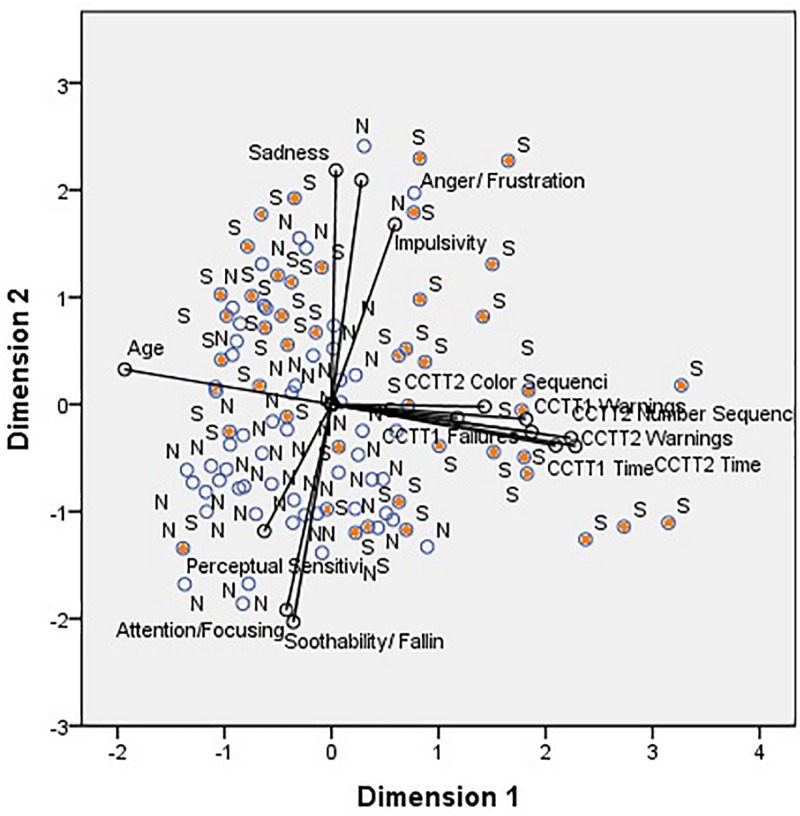
Principal component analysis performed on children from group S and group N. Cumulative percentage variance explained By Axes: I – 27.30%; I + II – 47.34%. Groups: S – Children who stutter; N – non-stuttering children. Variables: CCTT1 Time, CCTT1 failures, CCTT1 Warnings, CCTT2 Times, CCTT2 Warnings, CCTT2 number of sequencing errors, CCTT2 color sequencing errors, Anger/Frustration, Impulsivity, Sadness, Perceptual Sensitivity, Attention/Focusing, Soothability/Falling Reactivity, and age.

The PCA ordination biplot (Figure 1) showed that CCTT2 Time (component loading = 0.82), CCTT2 warnings (component loading = 0.80), CCTT1 time (component loading = 0.75), age (component loading = −0.69), CCTT2 number of sequencing errors (component loading = 0.67), CCTT1 warnings (component loading = 0.65), and CCTT2 Color sequencing errors (component loading = 0.51), were the variables influencing the children’s ordination along the first axis (Dimension 1), that is, the EF dimensions (Figure 1).

The right side of the axis shows the children with higher values of CCTT2 Time, CCTT2 warnings, CCTT1 time, CCTT2 number of sequencing errors, CCTT1 warnings, CCTT2 Color sequencing errors, and younger children. The left side of the axis shows children characterized by lower values of CCTT2 Time, CCTT2 warnings, CCTT1 time, CCTT2 number of sequencing errors, CCTT1 warnings, CCTT2 Color sequencing errors, and older children. Most of the children who stutter (“S”) were plotted on the right side of the first dimension. The first axis accounted for 27.30% of the total variance. The parameters with greater contribution to the second axis (dimension 2 – Temperament dimensions) were Sadness (component loading = 0.78), Anger/Frustration (component loading = 0.75), Soothability/Falling Reactivity (component loading = −0.72), Attention/Focusing (component loading = −0.69), and Impulsivity (component loading = 0.60). Most of the children who stutter were displayed on the upper part of the diagram, as they exhibited higher values of Sadness, Anger/Frustration and Impulsivity, and lower values of Soothability/Falling Reactivity and Attention/Focusing. The bottom part of the diagram shows mainly children who do not stutter, due to lower values of Sadness, Anger/Frustration and Impulsivity, and higher values of Soothability/Falling Reactivity and Attention/Focusing.

The second axis accounted for 20.04% of the total variance. The CCTT1 Time, CCTT1 Warnings, CCTT1 failures, CCTT2 Time, CCTT2 Warnings, CCTT2 number of sequencing errors, and CCTT2 color sequencing errors were highly and positively correlated with one another and negatively correlated with age. Sadness, Anger/Frustration, and Impulsivity were highly and positively correlated with each other; Attention, Soothability/Falling Reactivity and Perceptual Sensitivity were negatively correlated with Anger/Frustration, Impulsivity, and Sadness.

## Discussion

This study investigated temperament dimensions, EF skills, and anxiety levels in children who stutter and their non-stuttering peers. The main results are consistent with the hypothesis that some children who stutter may differ in temperament and EF factors when compared to children do not stutter. Specifically, in these group comparisons, children who stutter were found to be more reactive and sensitive than their non-stuttering peers. However, the findings were different across the two age groups that were analyzed. The differences in temperament level were noted in the group of older children only, while differences in EF were noted in the group of younger children only. Furthermore, results did not support the idea that children who stutter exhibit higher rates of anxiety than children who do not stutter, regardless of age group. Correlation analyses highlighted the dynamic nature of stuttering and suggested a link between endogenous abilities and external factors (Wolfe and Bell, 2004; Sudikoff et al., 2015).

### Temperament

Results on the temperament scale are consistent with previous studies that have suggested difficulties in children who stutter compared to non-stuttering peers in attention span (Embrechts et al., 2000; Anderson et al., 2003; Eggers et al., 2010; Costelloe et al., 2015; Hollister, 2015) and a tendency toward impulsivity (Schwenk et al., 2007; Eggers et al., 2013). Attention and impulsiveness suggested a link to emotion regulation (Rothbart et al., 2001), because negative levels suggest emotional instability (Derryberry and Rothbart, 1988; Eisenberg et al., 1993). We also found differences in Anger/Frustration, Sadness, and Soothability/Falling Reactivity temperament dimensions. This supports studies that indicate a more sensitive temperament in children who stutter. This could mean that school-age children who stutter may have more difficulty regulating their emotions. Furthermore, sadness could be connected to a more negative mood for children who stutter (Howell et al., 2004). A reactive temperament in children who stutter was also found in studies with preschoolers (Johnson et al., 2010; Ntourou et al., 2013) and school age children (Fowlie and Cooper, 1978). Higher scores in Anger/Frustration and lower scores in Soothability/Falling Reactivity could indicate that older children who stutter (ages 10–12 years) can have more difficulty in recovering from peak distress, excitement, or general arousal (i.e., they may have a harder time settling down after an exciting activity) (e.g., Karrass et al., 2006).

### Executive Functioning

Younger children who stutter required longer execution times and had a higher number of warnings and failures, number sequencing errors, and color sequencing errors compared age-matched peers who do not stutter. This suggests that children who stutter in the first years of schooling might have a lower attention span than their peers (Anderson et al., 2003). They might also need more time to adapt to a task and to start performing (Eggers et al., 2013; Manning and Beck, 2013) or have a greater concern about errors (Eichorn et al., 2017). A higher number of failures (times when a child almost makes a mistake) may be related to the tendency for impulsivity or difficulties with inhibitory control, as has been previously suggested by some authors (Schwenk et al., 2007; Eggers et al., 2013; Ofoe et al., 2018). This was especially true for the task requiring the alternation of colors in the sequence of numbers.

### Anxiety

No significant differences were detected between children who stutter and children who do not stutter in anxiety levels for either age group. According to previous studies, anxiety tends to increase as children grow older, especially between 8 to 12 years old (Blood and Blood, 2007; Messenger et al., 2015). These results are in agreement with prior researchers who reported no elevated anxiety in children who stutter (Mulcahy et al., 2008; Ortega and Ambrose, 2011; Smith et al., 2017). It could be that the participants in this study as a group showed no differences in anxiety because 22% were in speech therapy and another 22% had previously received treatment. Prior research has shown that people who are in or who have completed treatment often show comparable anxiety levels to their non-stuttering peers (Davis et al., 2002). Thus, balanced results between groups could be a consequence of the treatment itself. Other explanations may be due to methodological limitations, such as the lack of specificity of the measure to identify anxiety in the targeted population. As we saw above, anxiety in stuttering may be related to very specific situations, so, the use of a *trait* anxiety measure could have influenced the results. Speech tasks can trigger anxiety, so future research may benefit from using speech tasks rather than questionnaires (Manning and Beck, 2013; Gawda and Szepietowska, 2016). Finally, in self-report measures, children may try to give their answers a better view of themselves, trying to hide some perceived weaknesses and thereby under-reporting anxiety (Messenger et al., 2015).

### Temperament, Executive Functioning, and Anxiety Interaction

Looking closely at the differences between groups, it was possible to observe different results in the older participants through the parent-perception scale and in the younger participants through the performance on the EF task. It is hypothesized that Attention/Focusing, Perceptual Sensitivity, and Impulsivity issues may be subtle and unnoticed by the parents of the youngest children. Such differences may only be identifiable using sophisticated assessments such as the CCTT. In fact, some researchers agree that it is possible to find different results from behavioral measures (e.g., in novel events) and from parent reports of daily observations (Karrass et al., 2006). Moreover, parent perspectives may not reflect children’s true abilities (Bernstein Ratner and Silverman, 2000), because their responses may be influenced by the emotional link that exists with children (Seifer et al., 2004). Parents may also find it easier to identify temperament characteristics as children grow older, leading to more detailed or accurate assessment of children’s temperament in the older age group. The results should be interpreted with caution since the sample was not matched by gender, with sex differences being related to the fact that more females were found in the schools where the sample collection, of children who stutter, was carried out. Finally, many tasks with different sensory modalities can also influence the results (Ofoe et al., 2018). In the present study, EF was assessed using a visual search task, but for the temperament results, parents may have based their responses on situations that are dependent on other stimuli.

Because temperament characteristics can change over time (Rothbart et al., 2000), the different pattern between two age groups in temperament dimensions could also be related to the experience of negative emotional reactions and difficulties in functional communication abilities over time (Yaruss and Quesal, 2004; Yaruss, 2010). Current results from questionnaires may indicate that parents’ responses are affected by experiences rather than an inherent tendency. As older children become more aware of their stuttering, by experiencing it in different situations, they may experience greater impact of stuttering in their lives. This might exacerbate or emphasize certain characteristics to the parents’ view. When correlating the various components of temperament, EF, and anxiety, it was found that, difficulties in Attention/Focusing and Soothability/Falling Reactivity were correlated with a tendency toward greater sadness and Anger/Frustration. Results are in agreement with previous literature (Wolfe and Bell, 2004; Sudikoff et al., 2015) which suggests an association between the coordination and integration of mental processes in successful task performance with self-regulation of emotional states (Sudikoff et al., 2015).

### Temperament, Executive Functioning, and Anxiety Interaction and the DD-S Model

Findings from the current study support the predictions from the DD-S Model (Walden et al., 2012), which state that cognitive and emotional regulation can be activated by exogenous contexts. According to the model, the cause of stuttering moments is dynamic and not just related to external factors; it also relates to how children cope with exogenous factors through endogenous abilities (Walden et al., 2012). Further research on this dynamic relationship may be a starting point for better understanding the development of stuttering and the production of individual instances of disfluency. The present study helps to further specify the predictions of the DD-S model by the potential contribution of temperament and EF as intrinsic sensitivities, which can be triggered and boosted by external agents to influence the emergence of disfluencies.

### Future Directions

Because endogenous capacities, such as temperament and EF, can change over time, and because exogenous factors, such as demands of the environment, may be different for each person, future research should examine the interactions between temperament and the development of EF both individually and over time. Similar studies that involve the analysis of several variables simultaneously may help to better explain the onset of anxiety in older children or other aspects of how stuttering – and reactions to stuttering – develop over time.

In future research, the use of multiple instruments would strengthen both the reliability and validity of these findings. For example, experimental methods that complement self-perception scales might allow the evaluation and analysis of child behavior in different situations. It would also be worthwhile to add inhibitory control and working memory tasks to better understand EF. These are the concepts that are encompassed in EF and have been examined independently in other studies (Wolfe and Bell, 2004; Oyoun et al., 2010; Eggers et al., 2013; Ntourou et al., 2017). The DD-S model predicts that emotional reactivity and emotion regulation influence the frequency and severity of stuttering in preschool-age children, so it would be appropriate for future research to examine these factors simultaneously.

Future studies should also employ a more balanced sample collection, with a more tight matching of groups in variables such as sex, age, and other relevant factors. Although this study involved a reasonable sample size, the participants were in different stages of treatment, and it is possible that participants’ treatment histories might have affected the results. Similarly, the presence of some differences in sex ratio and age between sub-groups of children who stutter and children who do not stutter suggest that these preliminary results should be interpreted with caution.

## Conclusion

Results highlight the potential role of emotional processes, temperament, and EF in the development of stuttering. Examining the cognitive and emotional skills of children who stutter across age groups can add further knowledge about stuttering. Ultimately, such knowledge may lead to refinements in clinical and educational practices. A principal outcome of this study is the finding that endogenous abilities in children who stutter may be different according to their age. Older participants were found to be more prone to difficulties in temperament dimensions, while younger participants exhibited predispositions for difficulties related to EF. This suggests that differences between children who stutter and children who do not stutter may be mediated by age and development. These results are in agreement with a dynamic view of the development of stuttering influenced by internal and external factors.

## Data Availability Statement

The datasets generated for this study are available on request to the corresponding author.

## Ethics Statement

This study received full approval by the Ethics Committee of the Institute of Health Sciences of Universidade Católica Portuguesa (register number 34/2017). Prior to their participation in this study, parents signed a written informed consent for themselves and their children. Consent also included permission for the researcher to record the child and the right for participants to withdraw from the study at any time was clarified.

## Author Contributions

MR collected the sample, conducted the analysis, and wrote and edited the manuscript. JY contributed to the study design, analysis, and writing, editing, and reviewing of the manuscript. JR designed and supervised the study, and contributed to the writing and reviewing of the manuscript.

## Conflict of Interest

The authors declare that the research was conducted in the absence of any commercial or financial relationships that could be construed as a potential conflict of interest.

## References

[B1] Affrunti N. W. Woodruff-Borden J. (2015). The associations of executive function and temperament in a model of risk for childhood anxiety. *J. Child Fam. Stud.* 24 715–724. 10.1007/s10826-013-9881-4

[B2] Alarcão I. Sarmento M. Portugal G. Afonso N. Gaspar T. Vasconcelos T. (2009). *A educação das crianças dos 0 aos 12 anos.* Lisboa: Conselho Nacional de Educação.

[B3] Alm P. A. (2014). Stuttering in relation to anxiety, temperament, and personality: review and analysis with focus on causality. *J. Fluency Disord.* 40 5–21. 10.1016/j.jfludis.2014.01.004 24929463

[B4] Alm P. A. Risberg J. (2007). Stuttering in adults: the acoustic startle response, temperamental traits, and biological factors. *J. Commun. Disord.* 40 1–41. 10.1016/j.jcomdis.2006.04.001 16814317

[B5] Ambrose N. G. Yairi E. Loucks T. M. Seery C. H. Throneburg R. (2015). Relation of motor, linguistic and temperament factors in epidemiologic subtypes of persistent and recovered stuttering: initial findings. *J. Fluency Disord*. 45 12–26. 10.1016/j.jfludis.2015.05.004 26117417 PMC4546885

[B6] Anderson J. D. Pellowski M. W. Conture E. G. Kelly E. M. (2003). Temperamental characteristics of young children who stutter. *J. Speech Lang. Hear. Res.* 46 1221–1233. 10.1016/j.bbi.2008.05.010 14575354 PMC1458369

[B7] Anderson J. D. Wagovich S. A. (2010). Relationships among linguistic processing speed, phonological working memory, and attention in children who stutter. *J. Fluency Disord*. 35 216–234. 10.1016/j.jfludis.2010.04.003.Relationships 20831969 PMC2939037

[B8] Andrews J. G. Harris M. M. (1964). *The syndrome of stuttering, by Gavin Andrews and Mary Harris with Roger Garside and David Kay.* London: The Spastics Society Medical Education and Information Unit in association with Heinemann Medical Books.

[B9] Andrews G. Hoddinott S. Craig A. Howie P. Feyer A.-M. Neilson M. (1983). Stuttering: A review of research findings and theories circa 1982. *J. Speech Lang. Hear. Res.* 48 222–246. 10.1044/jshd.4803.226 6353066

[B10] Bajaj A. (2007). Working memory involvement in stuttering: exploring the evidence and research implications. *J. Fluency Disord.* 32 218–238. 10.1016/j.jfludis.2007.03.002 17825670

[B11] Bernstein Ratner N. Silverman S. (2000). Parental perceptions of children’s stuttering onset. *J. Speech Lang. Hear. Res.* 43 1252–1263. 10.1044/jslhr.4305.1252 11063245

[B12] Blair C. Ursache A. (2011). *Handbook of self-regulation: Research, theory, and applications*, 3rd Edn. New York, NY: Guilford Press.

[B13] Blake B. Pope T. (2008). Developmental psychology: incorporating piaget’s and vygotsky’s theories in classrooms. *J. CrossDiscip. Educ.* 1 59–67.

[B14] Blood G. W. Blood I. M. (2007). Preliminary study of self-reported experience of physical aggression and bullying of boys who stutter: relation to increased anxiety. *Percept. Mot. Skills.* 104 1060–1066. 10.2466/pms.104.4.1060-1066 17879638

[B15] Cho S.-C. Kim H.-W. Kim B.-N. Shin M.-S. Yoo H. J. Kim J.-W. (2011). Are teacher ratings and parents ratings differently associated with children’s intelligence and cognitive performance?. *Psychiatry Investig.* 8 15–21. 10.4306/pi.2011.8.1.15 21519532 PMC3079181

[B16] Conture E. G. (2001). *Stuttering: Its Nature, Diagnosis, and Treatment.* Boston, MA: Allyn and Bacon.

[B17] Costelloe S. E. Cavenagh P. Davis S. (2015). Are there any differences in attention levels between children who stammer and children who do not stammer, and what are the implications for therapy? *Procedia Soc. Behav. Sci*. 193 300–301. 10.1016/j.sbspro.2015.03.280

[B18] Cox N. J. Seider R. A. Kidd K. K. (1984). Some environmental factors and hypotheses for stuttering in families with several stutterers. *J. Speech Lang. Hear. Res.* 27 543–548. 10.1044/jshr.2704.543 6521460

[B19] Craig A. (2014). Major controversies in fluency disorders: clarifying the relationship between anxiety and stuttering. *J. Fluency Disord*. 40 1–3. 10.1016/j.jfludis.2014.05.001 24929462

[B20] Craig A. Hancock K. (1996). Anxiety in children and young adolescents who stutter. *Aust. J. Hum. Commun. Disord.* 24 28–38. 10.3109/asl2.1996.24.issue-1.04

[B21] Craig A. Hancock K. Tran Y. Craig M. (2003). Anxiety levels in people who stutter. *J. Speech Lang. Hear. Res.* 46 1197–1206. 10.1044/1092-4388(2003/093) 14575352

[B22] Craig A. Hancock K. Tran Y. Craig M. Peters K. (2002). Epidemiology of stuttering in the community across the entire life span. *J. Speech Lang. Hear. Res.* 45 1097–1105. 10.1044/1092-4388(2002/088) 12546480

[B23] Craig A. Tran Y. (2014). Trait and social anxiety in adults with chronic stuttering: conclusions following meta-analysis. *J. Fluency Disord.* 40 35–43. 10.1016/j.jfludis.2014.01.001 24929465

[B24] Craske M. G. Rauch S. L. Ursano R. Prenoveau J. Pine D. S. Zinbarg R. E. (2009). What is an anxiety disorder? *Depress. Anxiety.* 26 1066–1085. 10.1002/da.20633 19957279

[B25] Davis E. P. Bruce J. Gunnar M. R. (2002). The anterior attention network: associations with temperament and neuroendocrine activity in 6-year-old children. *Dev. Psychobiol.* 40 43–56. 10.1002/dev.10012 11835150

[B26] Davis S. Shisca D. Howell P. (2007). Anxiety in speakers who persist and recover from stuttering. *J. Commun. Disord.* 40 398–417. 10.1016/j.jcomdis.2006.10.003 17157866

[B27] Derryberry D. Rothbart M. K. (1988). Arousal, affect, and attention as components of temperament. *J. Pers Soc. Psychol.* 55 958–966. 10.1037/0022-3514.55.6.958 3216290

[B28] Diamond A. (2013). Executive functions. *Annu. Rev. Psychol.* 64 135–168. 10.1146/annurev-psych-113011-143750 23020641 PMC4084861

[B29] Donaher J. Richels C. (2012). Traits of attention deficit/hyperactivity disorder in school-age children who stutter. *J. Fluency Disord* 37 242–252. 10.1016/j.jfludis.2012.08.002 23218208

[B30] Druker K. Hennessey N. Mazzucchelli T. Beilby J. (2019). Elevated attention deficit hyperactivity disorder symptoms in children who stutter. *J. Fluency Disord.* 59 80–90. 10.1016/j.jfludis.2018.11.002 30477807

[B31] Eggers K. De Nil L. F. Van Den Bergh B. R. H. (2010). Temperament dimensions in stuttering and typically developing children. *J. Fluency Disord*. 35 355–372. 10.1016/j.jfludis.2010.10.004 21130269

[B32] Eggers K. De Nil L. F. Van den Bergh B. R. H. (2012). The efficiency of attentional networks in children who stutter. *J. Speech Lang. Hear. Res.* 55 946–959. 10.1044/1092-4388(2011/10-0208) 22232392

[B33] Eggers K. De Nil L. F. Van Den Bergh B. R. H. (2013). Inhibitory control in childhood stuttering. *J. Fluency Disord*. 38 1–13. 10.1016/j.jfludis.2012.10.001 23540909

[B34] Eichorn N. Marton K. Pirutinsky S. (2017). Cognitive flexibility in preschool children with and without stuttering disorders. *J. Fluency Disord.* 57 37–50. 10.1016/j.jfludis.2017.11.001 29157666

[B35] Eisenberg N. Fabes R. A. Bernzweig J. Karbon M. Poulin R. Hanish L. (1993). The relations of emotionality and regulation to preschoolers’ social skills and sociometric status. *Child Dev.* 64 1418–1438. 10.2307/1131543 8222881

[B36] Embrechts M. Ebben H. Franke P. Van de Poel C. (2000). “Temperament: A comparison between children who stutter and children who do not stutter,” in *Proceedings of the Third World Congress on Fluency Disorders: Theory, research, treatment, and self-he*, eds Bosshardt H. Yaruss J. HFM P., (Nijmegen: University of Nijmegen Presspp), 557–562.

[B37] Ezrati-Vinacour R. Levin I. (2004). The relationship between anxiety and stuttering: a multidimensional approach. *J. Fluency Disord.* 29 135–148. 10.1016/j.jfludis.2004.02.003 15178129

[B38] Felsenfeld S. Kirk K. Zhu G. Statham D. Neale M. Martin N. (2000). A study of the genetic and environmental etiology of stuttering in a selected twin sample. *Behav. Genet.* 30 359–366. 11235981 10.1023/a:1002765620208

[B39] Fowlie G. M. Cooper E. B. (1978). Traits attributed to stuttering and nonstuttering children by their mothers. *J. Fluency Disord.* 3 233–246. 10.1016/0094-730x(78)90023-2

[B40] Gawda B. Szepietowska E. (2016). Trait anxiety modulates brain activity during performance of verbal fluency tasks. *Front Behav Neurosci.* 10:10. 10.3389/fnbeh.2016.00010 26903827 PMC4748034

[B41] Goldsmith H. H. Buss A. H. Plomin R. Rothbart M. K. Thomas A. Chess S. (1987). Roundtable: what is temperament? Four approaches. *Child Dev.* 5 505–529. 10.2307/1130527 3829791

[B42] Guimarães I. (2007). *A ciência e a arte da voz humana.* Alcabideche: Escola Superior de Saúde de Alcoitão.

[B43] Guimarães I. Abberton E. (2005). Fundamental frequency in speakers of Portuguese for different voice samples. *J. Voice.* 19 592–606. 10.1016/j.jvoice.2004.11.004 16301105

[B44] Guitar B. (2014). *Stuttering: An Integrated Approach to its Nature and Treatment*, 4th Edn Baltimore, MD: Lippincott Williams & Wilkins.

[B45] Gupta R. Koscik T. R. Bechara A. Tranel D. (2011). The amygdala and decision making. *Neuropsychologia* 49 760–766. 10.1016/j.neuropsychologia.2010.09.029.The 20920513 PMC3032808

[B46] Hedge M. N. (1972). Stuttering, neuroticism and extroversion. *Behav. Res. Ther.* 10 395–397. 10.1016/0005-7967(72)90062-9 4637495

[B47] Heitmann R. R. Asbjørnsen A. Helland T. (2004). Attentional functions in speech fluency disorders. *Logoped. Phoniatr. Vocol.* 29 119–127. 10.1080/14015430410017379 15370643

[B48] Hollister J. E. (2015). *Effortful Control and Adaptive Functioning in School-Age Children Who Stutter.* Iowa, IA: University of Iowa.

[B49] Howell P. Davis S. Patel H. Cuniffe P. Downing-Wilson E. Au-Yeung J. (2004). “Fluency development and temperament in fluent children and children who stutter,” in *Theory, Research and Therapy in Fluency Disorders*, eds Packman A. Meltzer A. Peters H. F. M., (Nijmegen: Nijmegen University Press), 250–256.

[B50] Iverach L. Lowe R. Jones M. Brian S. O. Menzies R. G. Packman A. (2017). A speech and psychological profile of treatment-seeking adolescents who stutter. *J. Fluency Disord.* 51 24–38. 10.1016/j.jfludis.2016.11.001 28212718

[B51] Iverach L. Menzies R. G. Brian S. O. Packman A. Onslow M. (2011). Anxiety and stuttering: continuing to explore a complex relationship. *Am. J. Speech Lang. Pathol.* 20 221–233. 10.1044/1058-0360(2011/10-0091) 21478283

[B52] Johnson K. N. Walden T. A. Conture E. G. Karrass J. (2010). Spontaneous regulation of emotions in preschool children who stutter: preliminary findings. *J. Speech Lang. Hear. Res.* 53 1478–1495. 10.1044/1092-4388(2010/08-0150) 20643793 PMC3800203

[B53] Jones R. Choi D. Conture E. Walden T. (2014). Temperament, emotion and childhood stuttering. *Semin. Speech Lang.* 35 114–131. 10.1055/s-0034-1371755 24782274 PMC4317269

[B54] Kaganovich N. Wray A. H. Weber C. (2010). Non-linguistic auditory processing and working memory update in pre- school children who stutter: an electrophysiological study. *Dev. Neuropsychol.* 35 712–736. 10.1080/87565641.2010.508549 21038162 PMC3059510

[B55] Karrass J. Walden T. A. Conture E. G. Graham C. G. Arnold H. S. (2006). Relation of emotional reactivity and regulation to childhood stuttering. *J. Commun. Disord.* 39 402–423. 10.1016/j.jfludis.2012.12.004 16488427 PMC1630450

[B56] Kefalianos E. Onslow M. Block S. Menzies R. Reilly S. (2012). Early stuttering, temperament, and anxiety: Two hypotheses. *J. Fluency Disord.* 37 151–163. 10.1016/j.jfludis.2012.03.002 22682317

[B57] Kennel S. Taylor A. G. Lyon D. Bourguignon C. (2010). Pilot feasibility study of binaural auditory beats for reducing symptoms of inattention in children and adolescents with attentiondeficit/hyperactivity disorder. *J. Pediatr. Nurs.* 25 3–11. 10.1016/j.pedn.2008.06.010 20117669

[B58] Konstantopoulos K. Vogazianos P. Thodi C. Nikopoulou-Smyrni P. (2015). A normative study of the children’s color trails test (CCTT) in the cypriot population. *Child Neuropsychol*. 21 751–758. 10.1080/09297049.2014.924491 24898762

[B59] Koo H. J. Min S. S. (2008). A standardization study of children’s color trails test (CCTT). *J. Korean Acad. Child Adolesc. Psychiatry* 19 28–37.

[B60] Llorente A. M. Voigt R. G. Williams J. Frailey J. K. Satz P. D’Elia L. F. (2009). Children’s color trails test 1 2: test-retest reliability and factorial validity. *Clin. Neuropsychol.* 23 645–660. 10.1080/13854040802427795 18942031

[B61] Llorente A. M. Williams J. S. D’Elia L. F. (2003). *Children’s Color Trails Test: Professional Manual.* Lutz, FL: Psychological Assessment Resources.

[B62] Manning W. Beck J. G. (2013). The role of psychological processes in estimates of stuttering severity. *J. Fluency Disord.* 38 356–367. 10.1016/j.jfludis.2013.08.002 24331243

[B63] March J. S. Parker J. D. A. Sullivan K. Stallings P. Conners C. K. (1997). The multidimensional anxiety scale for children (MASC): factor structure, reliability, and validity. *J. Am. Acad. Child. Adolesc. Psychiatry.* 36 554–565. 10.1097/00004583-199704000-00019 9100431

[B64] Matos M. G. Gina T. Borges A. I. Manso D. Simões C. Ferreira A. (2012). Anxiety, depression and coping: CDI, MASC and CRI-Y for screening purposes in schools. *Span. J. Psychol.* 15 348–356. 10.5209/rev_SJOP.2012.v15.n1.37341 22379724

[B65] McAllister J. Kelman E. Millard S. (2015). Anxiety and cognitive bias in children and young people who stutter. *Procedia Soc. Behav. Sci.* 193 183–191. 10.1016/j.sbspro.2015.03.258

[B66] Messenger M. Packman A. Onslow M. Menzies R. O’Brian S. (2015). Children and adolescents who stutter: further investigation of anxiety. *J. Fluency Disord.* 46 15–23. 10.1016/j.jfludis.2015.07.006 26292910

[B67] Miyake A. Friedman N. P. Emerson M. J. Witzki A. H. Howerter A. Wager T. D. (2000). The unity and diversity of executive functions and their contributions to complex “Frontal Lobe” tasks: a latent variable analysis. *Cogn. Psychol.* 41 49–100. 10.1006/cogp.1999.0734 10945922

[B68] Moon K. R. Chung S. M. Park H. S. Kim H. S. (2012). Materials of acoustic analysis: sustained vowel versus sentence. *J. Voice.* 26:5 563–565. 10.1016/j.jvoice.2011.09.007 22516312

[B69] Mulcahy K. Hennessey N. Beilby J. Byrnes M. (2008). Social anxiety and the severity and typography of stuttering in adolescents. *J. Fluency Disord.* 33 306–319. 10.1016/j.jfludis.2008.12.002 19328982

[B70] Nicholas A. Yairi E. Mangelsdorf S. Jiang M. Cook F. (2015). The temperament of school aged children who stutter: their view. *Procedia Soc. Behav. Sci*. 193 323–324. 10.1016/j.sbspro.2015.03.296

[B71] Nigg J. T. (2000). On inhibition/disinhibition in developmental psychopathology: views from cognitive and personality psychology and a working inhibition taxonomy. *Psychol. Bull.* 126 220–246. 10.1037/0033-2909 10748641

[B72] Ntourou K. Anderson J. D. Wagovich S. A. (2017). Executive function and childhood stuttering: parent ratings and evidence from a behavioral task. *J. Fluency Disord.* 56 18–32. 10.1016/j.jfludis.2017.12.001 29443692 PMC5970042

[B73] Ntourou K. Conture E. G. Walden T. A. (2013). Emotional reactivity and regulation in preschool-age children who stutter. *J. Fluency Disord.* 38 260–274. 10.1016/j.jfludis.2013.06.002 24238388 PMC3834351

[B74] Ofoe L. C. Anderson J. D. Ntourou K. (2018). Short-term memory, inhibition, and attention in developmental stuttering: a meta-analysis. *J. Speech Lang. Hear. Res.* 61 1626–1648. 10.1044/2018_JSLHR-S-17-0372 29984373 PMC6195058

[B75] Ortega A. Y. Ambrose N. G. (2011). Developing physiologic stress profiles for school-age children who stutter. *J. Fluency Disord.* 36 268–273. 10.1016/j.jfludis.2011.04.007 22133403

[B76] Oyler M. E. (1996). Temperament: Stuttering and the behaviorially inhibited child. *Paper Presented in the Annual Convention of the American Speech - Language - Hearing Association*, Seattle, WA.

[B77] Oyoun H. A. El Dessouky H. Shohdi S. Fawzy A. (2010). Assessment of working memory in normal children and children who stutter. *J. Am. Sci.* 6 562–569.

[B78] Peters H. F. M. Hulstijn W. (1984). Stuttering and anxiety: the difference between stutterers and nonstutterers in verbal apprehension and physiologic arousal during anticipation of speech and non-speech tasks. *J. Fluency Disord.* 9 67–84. 10.1016/0094-730x(84)90008-1

[B79] Pinto A. B. (2008). *Desenvolvimento das funções executivas em crianças dos 6 aos 11 anos de idade.* Porto: Universidade do Porto.

[B80] Reilly S. Onslow M. Packman A. Wake M. Bavin E. Prior M. (2009). Predicting stuttering onset by the age of 3 years: a prospective, community cohort study. *Pediatrics*. 123 270–277. 10.1542/peds.2007-3219 19117892 PMC3879585

[B81] Riley G. (2009). *The Stuttering Severity Instrument for Adults and Children (SSI-4)*, 4th Edn Austin, TX: PRO-ED.

[B82] Riley J. Riley G. (2000). A revised component model for diagnosing and treating children who stutter. *Contemp. Issues Commun. Sci. Disord.* 27 188–199. 10.1044/cicsd_27_F_188

[B83] Rocha M. Rato J. R. (2017). *Questionário de Temperamento na Terceira Infância: European Portuguese Version of the Temperament in Middle Childhood Questionnaire (Phd project).* Lisboa: Universidade Católica Portuguesa.

[B84] Rothbart M. K. Ahadi S. A. Evans D. E. (2000). Temperament and personality: origins and outcomes. *J. Pers. Soc. Psychol.* 78 122–135. 10.1037/0022-3514.78.1.122 10653510

[B85] Rothbart M. K. Ahadi S. A. Hershey K. L. Fisher P. (2001). Investigations of temperament at three to seven years: the children’s behavior questionnaire. *Child Dev.* 72 1394–1408. 10.1111/1467-8624.00355 11699677

[B86] Rothbart M. K. Hwang J. (2002). Measuring infant temperament. *Infant Behav. Dev.* 25 113–116. 10.1016/S0163-6383(02)00109-1

[B87] Salvador M. C. Matos A. P. Oliveira S. March J. S. Arnarson E. Ö Carey S. (2017). A Escala Multidimensional de Ansiedade para Crianças (MASC): Propriedades psicométricas e análise fatorial confirmatória numa amostra de adolescentes Portugueses. *Re. Iberoam. Diagn. Ev.* 45:3 33–46. 10.21865/RIDEP45.3.03

[B88] Samochiş L. Lazãr S. Iftene F. (2011). Clinical aspects: aspects of the anxiety and depression at the stuttering child. *Acta Medica Transilvanica. II* 1 188–191.

[B89] Schwenk K. A. Conture E. G. Walden T. A. (2007). Reaction to background stimulation of preschool children who do and do not stutter. *J. Commun. Disord.* 40 129–141. 10.1016/j.jcomdis.2006.06.003 16876188 PMC4123446

[B90] Seifer R. Sameroff A. Dickstein S. Schiller M. Hayden L. C. (2004). Your own children are special: clues to the sources of reporting bias in temperament assessments. *Infant Behav. Dev.* 27 323–341. 10.1016/j.infbeh.2003.12.005

[B91] Silvestre I. (2009). *Avaliação Acústico-Perceptiva e Stress em Mulheres com Patologia Laríngea Inês dos Reis Silvestre Avaliação Acústico-Perceptiva e Stress em Mulheres com Patologia Laríngea.* Portugal: Universidade de Aveiro.

[B92] Silvestre I. Guimarães I. Teixeira A. (2011). Qualidade vocal em mulheres com diagnóstico de nódulos vocais: Estudo preliminar. *Rev. Bras. Otorrinolaringol.* 49 69–77.

[B93] Simonds J. (2006). *The Role of Reward Sensitivity and Response Execution in Childhood Extraversion.* Oregon: University of Oregon.

[B94] Simonds J. Rothbart M. (2004). *Temperament in Middle Childhood Questionnaire (Version 3.0).* Oregon: University of Oregon.

[B95] Singer J. M. Fagen J. W. (1992). Negative affect, emotional expression, and forgetting in young infants. *Dev. Psychol.* 28 48–57. 10.1037/0012-1649.28.1.48

[B96] Smith A. Weber C. (2017). How stuttering develops: the multifactorial dynamic pathways theory. *J. Speech Lang. Hear. Res.* 60 2483–2505. 10.1044/2017_JSLHR-S-16-0343 28837728 PMC5831617

[B97] Smith K. A. Iverach L. O’Brian S. Kefalianos E. Reilly S. (2014). Anxiety of children and adolescents who stutter: a review. *J. Fluency Disord.* 40 22–34. 10.1016/j.jfludis.2014.01.003 24929464

[B98] Smith K. A. Iverach L. O’Brian S. Mensah F. Kefalianos E. Hearne A. (2017). Anxiety in 11-year-old children who stutter: findings from a prospective longitudinal community sample. *J. Speech Lang. Hear. Res.* 60 1211–1222. 10.1044/2016_JSLHR-S-16-0035 28418529

[B99] Spaulding T. J. Plante E. Vance R. (2008). Sustained selective attention skills of preschool children with specific language impairment: evidence for separate attentional capacities. *J. Speech Lang. Hear. Res* 51 16–34. 10.1044/1092-4388(2008/002) 18230853

[B100] Sudikoff E. L. Bertolin M. Lordo D. N. Kaufman D. A. S. (2015). Relationships between executive function and emotional regulation in healthy children. *J. Neurol. Psychol.* S, 8.

[B101] Thomas A. Chess S. (1996). *Temperament Theory and Practice.* New York, NY: Brunner/Mazel Publishers.

[B102] van der Merwe B. Robb M. P. Lewis J. G. Ormond T. (2011). Anxiety measures and salivary cortisol responses in preschool children whos stutter. *Contemp. Issues Commun. Sci. Disord.* 38 1–10. 10.1044/cicsd_38_s_1

[B103] Walden T. A. Frankel C. Buhr A. Johnson K. Karrass J. M. (2012). Contributions to developmental stuttering. *J. Abnorm. Child Psychol.* 40 633–644. 10.1007/s10802-011-9581-8.Dual 22016200 PMC3740566

[B104] Wei C. Hoff A. Villabo M. Peterman J. McCracken J. Walkup J. (2014). Assessing anxiety in youth with the multidimensional anxiety scale for children (MASC). *J. Clin. Child Adolesc. Psychol*. 43 566–578. 10.1080/15374416.2013.814541 23845036 PMC3858516

[B105] Williams J. Rickert V. Hogan J. Zolten A. J. Satz P. D’elia L. F. (1995). Children’s color trails. *Arch. Clin. Neuropsychol.* 10 211–223. 10.1016/0887-6177(94)00041-n 14588688

[B106] Wolfe C. D. Bell M. A. (2004). Working memory and inhibitory control in early childhood: contributions from physiology, temperament, and language. *Dev. Psychobiol.* 44 68–83. 10.1002/dev.10152 14704991

[B107] Yairi E. Ambrose N. G. (2005). *Early Childhood Stuttering.* Texas, TX: Pro-Ed.

[B108] Yaruss J. S. (2010). Assessing quality of life in stuttering treatment outcomes research. *J. Fluency Disord.* 35 190–202. 10.1016/j.jfludis.2010.05.010 20831967

[B109] Yaruss J. S. Quesal R. W. (2004). Stuttering and the international classification of functioning, disability, and health (ICF): an update. *J. Commun. Disord.* 37 35–52. 10.1016/S0021-9924(03)00052-2 15013378

